# Subjective Household Economic Status and Obesity in Toddlers: A Cross-Sectional Study of Daycare Centers in Japan

**DOI:** 10.2188/jea.JE20170081

**Published:** 2019-01-05

**Authors:** Yasutake Tomata, Kumiko Tanno, Shu Zhang, Michiko Sakai, Kaori Kobayashi, Noriko Kurasawa, Miki Tanaka, Yuka Kamada, Ichiro Tsuji, Fukuko Hiramoto

**Affiliations:** 1 Division of Epidemiology, Department of Health Informatics and Public Health, Tohoku University School of Public Health, Graduate School of Medicine, Miyagi, Japan; 2 Department of Health and Nutrition, Faculty of Food and Nutrients, Miyagi Gakuin Women’s University and Graduate School, Miyagi, Japan; 3 Course of Health and Nutrition, Miyagi Gakuin Women’s University Graduate School of Health and Nutrition, Miyagi, Japan; 4 Food Service Research Committee, Sendai Association of Daycare Centers, Miyagi, Japan

**Keywords:** economic status, obesity, Japan, children

## Abstract

**Background:**

Although lower household economic status is known to be a risk factor for obesity among school-age children, such an association among toddlers remains unclear. The present study investigated the association between household economic status and obesity in toddlers.

**Design:**

We conducted a cross-sectional study of children aged 4 years attending daycare centers in Japan. Information on subjective household economic status [“affluent”, “neither”, “less affluent”, or “non-affluent”] was collected via questionnaire from the children’s guardians in 2015. Based on measured values of height and weight, obesity was defined using the International Obesity Task Force cut-offs of overweight (BMI ≥17.47 for boys and ≥17.19 for girls). We used the logistic regression model to investigate the association between household economic status and obesity.

**Results:**

Among 1,848 respondents, the prevalence of obesity was 6.8%. Non-affluent household economic status was associated with a significantly higher probability of obesity in toddlers; the multivariate adjusted odds ratio for “non-affluent” households was 2.31 (95% confidence interval, 1.23–4.33) compared with “affluent” households.

**Conclusion:**

Perception of non-affluent economic status by the guardian was associated with a higher probability of toddler obesity. This result suggests that non-affluent household economic status is associated with obesity in toddlers.

## INTRODUCTION

The prevalence of obesity in toddlers has increased globally.^1^ In the Asia Pacific Region, including Japan, malnutrition in children has markedly decreased, whereas obesity is now a growing public health problem.^2^

In western countries, lower socioeconomic status is known to be a risk factor for obesity among school-age children.^1^^,^^3^ Additionally, socioeconomic inequalities in obesity prevalence continue to widen in western countries.^3^

Income inequality in Japan (Gini coefficient) is relatively higher than in other Organization for Economic Cooperation and Development (OECD) countries, and it has increased in the period from 1985 to 2011.^4^ Child income poverty rates are also relatively higher than in other OECD countries.^5^ Therefore, there is a concern that lower socioeconomic status in Japan may also affect the prevalence of childhood obesity.^5^^–^^7^ Indeed, recent Japanese cross-sectional studies have reported that lower household economic status (income or subjective economic status) is a risk factor for obesity in adolescents.^8^^,^^9^ This increased risk may be problematic not only in school-age children, but also at earlier life-stages (ie in pre-school children). Despite these concerns, the association between household economic status and obesity in toddlers has not been clarified.

The aim of the present analysis was to investigate the relationship between household economic status and obesity in toddlers in Japan.

## METHODS

### Study design

This cross-sectional study was conducted between October and December, 2015. The study subjects comprised all 4-year-old children (54–68 months old) who attended government-authorized daycare centers in Sendai city: 2,738 boys and girls from 143 daycare centers. The Sendai Association of Daycare Centers requested cooperation of the dietitian at each daycare center in the questionnaire survey. The dietitian distributed the questionnaire to the guardians of the children. The survey included questions about subjective household economic status, time affluence (having spare time), and health literacy of the guardian. For height and weight of children, the latest assessment values were transcribed from each daycare center record. The dietitian at each daycare center input these collected data into the specified form of the Sendai Association of Daycare Centers, thus providing an anonymous dataset.

### Participants

**Figure 1.**
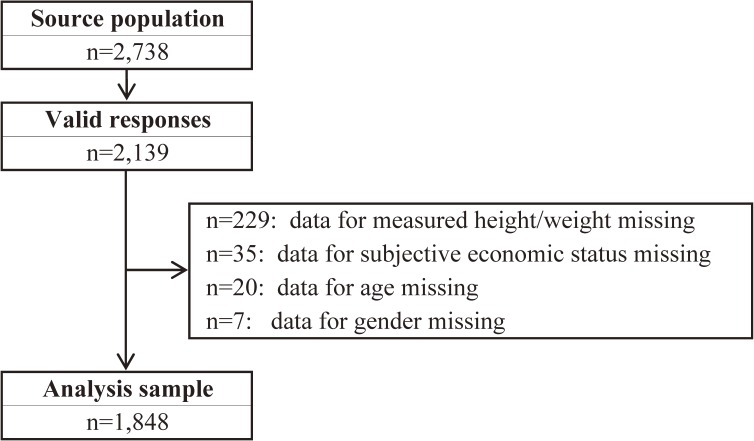
Flow chart of study participants

Among the 2,738 subjects, 2,139 provided valid responses (95.4% of respondents were the mother, 3.8% were the father, and 0.8% were others). We excluded 229 subjects for whom measured height/weight data were missing, 35 for whom data on subjective household economic status were missing, 20 for whom data on age were missing, and 7 for whom data on gender were missing (Figure 1). Thus, a total of 1,848 subjects were analyzed for the purpose of this study.

### Exposure

Subjective household economic status was assessed by asking the question “How do you feel about your current household economic situation?”, for which available responses were: “most affluent”, “more affluent”, “neither more nor less” (named “neither”), “less affluent”, or “non-affluent”. This question was the same as the question in the National Nutrition Survey of Preschool Children (Ministry of Health, Labor and Welfare).^10^ Subjective household economic status has been used to assess poverty status in preliminary research.^9^^,^^11^^,^^12^ Subjective household economic status is an indicator of satisfaction with one’s life situation or with life domains, such as income, health, leisure time, environment, or social integration.^13^ In a previous study to compare the explanatory power of objective and subjective economic status on perceived life quality measures, subjective economic status accounted for more variance in life quality measures than did objective economic status (income).^14^

To focus on childhood poverty, we combined the “most affluent” (7.2%) and “more affluent” (22.4%) categories into a single one: “affluent” (29.6%). Thus, the subjects were grouped into four categories: 1) “affluent”, 2) “neither”, 3) “less affluent”, and 4) “non-affluent”.

### Outcome

Obesity was defined according to the BMI value cut-offs for overweight established by the International Obesity Task Force (boy: ≥17.47 kg/m^2^, girl: ≥17.19 kg/m^2^).^8^^,^^15^

### Covariates

Time affluence (having spare time) was assessed by asking the question “How do you feel about your current time affluence?”, for which available responses were: “most affluent”, “more affluent”, “neither more nor less”, “less affluent”, or “non-affluent”.^10^ In the same way as for subjective household economic status, we grouped the subjects into four categories: “affluent”, “neither”, “less affluent”, and “non-affluent”.

The Communicative and Critical Health Literacy scale was used as an indicator of health literacy.^16^ Using five questions, guardians were asked about three items for communicative health literacy and two items for critical health literacy. These questions asked whether the participant would be able to (1) collect health-related information from various sources, (2) extract the relevant information, (3) understand and communicate the obtained information, (4) consider the credibility of the information, and (5) make decisions based on the information, specifically in the context of health-related issues. A previous study reported that Cronbach’s α for the five items was 0.86.^16^ Each item was rated on a 5-point scale, and the total point score ranged from 5 to 25.^17^

### Ethical consideration

The study was approved by the Miyagi Gakuin Women’s University Ethics Review Committee (No. 2015-3) in Sendai, Japan. Only anonymous data were available for the present study.

### Statistical analysis

We used the multiple adjusted logistic regression model to calculate the odds ratios (ORs) and 95% confidence intervals (CIs) for obesity according to the subjective household economic status categories, with “affluent” used as a reference category. Multivariate models were adjusted for the following variables: model 1 was gender- and age-adjusted, while model 2 was further adjusted for time affluence and health literacy score.

Moreover, we also conducted stratified analyses according to gender (boys or girls) and time affluence (“>less affluent” [“affluent”, “neither”] or “≤less affluent” [“less affluent”, “non-affluent”]). For these stratified analyses, neither of these factors was used as a respective covariate. Additionally, *P*-interactions were tested through addition of cross-product terms to model 2.

All the data were analyzed using IBM SPSS version 24 (IBM Software Group, Chicago, IL, USA). All statistical tests described here were two-sided, and differences at *P* < 0.05 were accepted as significant.

## RESULTS

### Characteristics

Table 1 compares the characteristics of participants according to subjective household economic status. Participants who considered themselves to have “non-affluent” household economic status tended to have “non-affluent” time affluence and lower health literacy (lower mean score).

**Table 1.** Characteristics according to subjective household economic status (*n* = 1,848)

	Subjective household economic status			

	Affluent	Neither	Less affluent	Non-affluent
*n*	547	600	536	165

Boy, %	50	49	50	48
Toddler’s age, months	61.5	61.4	61.3	61.6
Health literacy, points^a^	19.3	18.5	18.1	17.2
Time affluence, %^b^				
Affluent	28	16	15	8
Neither	16	27	14	9
Less affluent	46	47	55	46
Non-affluent	11	10	16	36

^a^The Communicative and Critical Health Literacy score of guardians.

^b^Time affluence (having spare time) of guardians was assessed by asking the question “How do you feel about your current time affluence?”.

### Economic status and obesity

The association between subjective household economic status and obesity, along with ORs and associated 95% CIs, is shown in Table 2. Even after addition of adjustment items, we found that households with “non-affluent” economic status were associated with a higher probability of toddler obesity; the multivariate OR was 2.31 (95% CI, 1.23–4.33) in model 2.

**Table 2.** Subjective household economic status and toddler obesity (*n* = 1,848)

	Total, *n*	Obesity		Model 1^a^	Model 2^b^
		
		*n*	%	OR (95% CI)	OR (95% CI)
Subjective household economic status					
Affluent	547	34	6.2	1.00 (reference)	1.00 (reference)
Neither	600	44	7.3	1.19 (0.75–1.90)	1.25 (0.78–2.01)
Less affluent	536	29	5.4	0.87 (0.52–1.45)	0.95 (0.56–1.60)
Non-affluent	165	19	11.5	1.95 (1.08–3.52)	2.31 (1.23–4.33)

*P*-trend				0.276	0.139

CI, confidence interval; OR, odds ratio.

^a^Model 1 was adjusted for gender and age (month; continuous).

^b^Model 2 was adjusted for gender, age (month; continuous), health literacy score (tertile categories: ≤18 points, 19–20 points, ≥21 points, missing), time affluence (more affluent, neither, less affluent, non-affluent, missing).

Even when the original variable of subjective household economic status was applied (“most affluent” [*n* = 133] being used as a reference category), we also found that households with “non-affluent” economic status tended to have a higher probability of toddler obesity; the multivariate OR was 2.02 (95% CI, 0.86–4.73) in model 2 (eTable 1).

### Stratified analysis

We conducted stratified analyses to check whether the association between subjective household economic status and obesity was altered by gender and time affluence. No significant interactions were observed in model 2 (Table 3). However, in the “≤less affluent” stratum (less affluent, non-affluent), the OR for “non-affluent” was significantly higher in model 2, and a significant dose-response relationship was observed (*P*-trend = 0.041).

**Table 3.** Subjective household economic status and obesity stratified by gender or time affluence (*n* = 1,848)

	Total, *n*	Obesity		Model 1^a^	Model 2^b^	*P*-interaction^b^
		
		*n*	%	OR (95% CI)	OR (95% CI)	
Gender						
Boys (*n* = 917)						
Affluent	276	15	5.4	1.00 (reference)	1.00 (reference)	
Neither	294	18	6.1	1.14 (0.56–2.31)	1.38 (0.66–2.87)	
Less affluent	267	12	4.5	0.82 (0.38–1.79)	1.02 (0.45–2.27)	
Non-affluent	80	8	10.0	1.93 (0.79–4.73)	2.61 (1.01–6.72)	
*P*-trend				0.524	0.222	0.872

Girls (*n* = 931)						
Affluent	271	19	7.0	1.00 (reference)	1.00 (reference)	
Neither	306	26	8.5	1.23 (0.67–2.28)	1.19 (0.63–2.23)	
Less affluent	269	17	6.3	0.91 (0.46–1.79)	0.91 (0.45–1.82)	
Non-affluent	85	11	12.9	1.97 (0.90–4.33)	2.16 (0.92–5.05)	
*P*-trend				0.372	0.348	

Time affluence						
>Less affluent time (Affluent, Neither) (*n* = 683)						
Affluent	239	15	6.3	1.00 (reference)	1.00 (reference)	
Neither	260	27	10.4	1.77 (0.91–3.42)	1.80 (0.92–3.53)	
Less affluent	155	7	4.5	0.71 (0.28–1.79)	0.68 (0.27–1.74)	
Non-affluent	29	2	6.9	1.11 (0.24–5.14)	1.08 (0.23–5.13)	
*P*-trend				0.746	0.651	0.191

≤Less affluent time (Less affluent, Non-affluent) (*n* = 1,162)						
Affluent	308	19	6.2	1.00 (reference)	1.00 (reference)	
Neither	339	17	5.0	0.80 (0.41–1.56)	0.82 (0.42–1.62)	
Less affluent	379	22	5.8	0.93 (0.50–1.76)	1.03 (0.54–1.97)	
Non-affluent	136	17	12.5	2.14 (1.07–4.26)	2.35 (1.16–4.76)	
*P*-trend				0.079	0.041	

CI, confidence interval; OR, odds ratio.

^a^Model 1 was adjusted for gender (except in the stratified analysis by gender) and age (month; continuous).

^b^Model 2 was adjusted for gender (except in stratified analysis by gender), age (month; continuous), health literacy score (tertile categories: ≤18 points, 19–20 points, ≥21 points, missing), time affluence (more affluent, neither, less affluent, non-affluent, missing; except in the stratified analysis by time affluence).

## DISCUSSION

In this cross-sectional study, we investigated the association between subjective household economic status and toddler obesity. We found that perception of non-affluent economic status by the guardian was associated with a significantly higher probability of toddler obesity. To our knowledge, this is the first academic report to have proved an association between household economic status and toddler obesity in Japan.

A previous Japanese study reported a higher probability of obesity in adolescents (12–18 years old) from low-income households, but this was not the case for children of elementary school age (6–11 years old).^8^ The authors suggested that the availability of school lunches might reduce the difference in the risk of obesity. In the present study, the subjects were toddlers who attended daycare centers; they were provided with not only lunch and snacks, but also had the opportunity to take part in physical activity and to receive dietary education under the management of the daycare center. The present participants were considered to have different exposures only when they were not at the daycare centers. Nevertheless, our data suggested a significant difference in the prevalence of obesity according to household economic status.

It is known that subjective economic status is correlated with income, although these do not directly overlap.^18^ Whereas income poverty is based on external criteria, subjective poverty is based on perceptions of external circumstances. For example, a previous study reported that subjective economic status was a more accurate predictor of unhealthy dietary intake for preschool children than was income.^19^ Based on this result, the authors considered the possibility that perception of ability to cope with income rather than income per se contributed to parental family food choices. Therefore, to understand the relationship between household economic status and childhood obesity, it is necessary to consider not only income but also subjective economic status.

It is also known that poor household economic status is related to insufficient knowledge and a negative attitude toward a healthy diet.^8^^,^^20^ In the present study, although health literacy scores tended to be lower in households with non-affluent economic status (Table 1), the results did not change substantially when adjustment for the health literacy score was performed (Table 2). Therefore, it was unlikely that health literacy would have largely explained this relationship.

In sensitivity analysis, we observed a significant relationship between subjective household economic status and obesity among subjects who did not have time affluence (Table 3). In terms of dietary habits, lower socio-economic status is related to greater access to energy-dense diets.^1^ Our preliminary study also suggested that subjective economic status was related to dietary behavior.^21^ If guardians have neither sufficient income nor time affluence, they might have a tendency to serve their children convenient processed food.^22^ However, there was a particularly small number (*n* = 29) of households with “non-affluent” economic status in the “>less affluent time” stratum, and the interaction was not statistically significant (*P*-interaction = 0.191). Therefore, in the present study, the modification effect of time affluence on the association between economic status and obesity remained unclear.

This study had several limitations. First, because the present analysis was cross-sectional in design, no temporal relationship between economic status and obesity can be inferred. Prospective studies will be required to establish a causal link between economic status and obesity. Second, some misclassification of exposure measurement and outcome measurement might have occurred. The present study adopted subjective household economic status, but did not include the amount of household income as the exposure. Although subjective assessment of household economic status is assumed to be useful,^9^^,^^23^ it is not an objective evaluation and not based on a quantitative cut-off value. Furthermore, the method for measurement of height and weight was not strictly defined (eg, instruments used, measurement date, or measurement time). Thus, measurement deviation might not have been minimized. If there had been a high degree of non-differential misclassification, the present results would have been underestimated.^24^ Third, detailed characteristics (eg, nutritional status during pregnancy) of the guardians were not investigated. Fourth, the results should be viewed in the social context of Japan, and might not be generalizable to other countries. In the present study, “non-affluent” economic status would not mainly reflect a state of absolute poverty in which it is difficult to obtain food (starvation). However, in some developing countries, “non-affluent” economic status may mainly reflect absolute poverty, and “non-affluent” economic status may not contribute to obesity.

In conclusion, this study suggests that non-affluent household economic status is associated with obesity among toddlers in Japan. Our findings imply that poor household economic status increases the risk of obesity in the early stage of childhood (pre-school age).

## APPENDIX A. SUPPLEMENTARY DATA

eTable 1. Subjective household economic status and toddler obesity (n = 1,848)
