# Supplementary material for: Subjective Household Economic Status and Obesity in Toddlers: A Cross-Sectional Study of Daycare Centers in Japan
Source: J Epidemiol. 2019 Jan 5;29(1):33–7. doi: 10.2188/jea.JE20170081 (PMC6290275; doi:10.2188/jea.JE20170081)
Supplement: Supplementary file 1 [file je-29-033-s001.pdf]

**eTable 1.** Subjective household economic status and toddler obesity (n=1,848)

| | Total, n | Obesity | | Model 1[a] | | Model 2[b] | |
|---|---|---|---|---|---|---|---|
| | | n | % | OR | (95% CI) | OR | (95% CI) |
| Subjective household economic status | | | | | | | |
| Most affluent | 133 | 10 | 7.5 | 1.00 (reference) | | 1.00 (reference) | |
| More affluent | 414 | 24 | 5.8 | 0.77 | (0.36–1.65) | 0.84 | (0.38–1.82) |
| Neither | 600 | 44 | 7.3 | 0.98 | (0.48–2.01) | 1.10 | (0.52–2.29) |
| Less affluent | 536 | 29 | 5.4 | 0.72 | (0.34–1.51) | 0.83 | (0.38–1.79) |
| Non-affluent | 165 | 19 | 11.5 | 1.61 | (0.72–3.59) | 2.02 | (0.86–4.73) |
| p-trend | | | | 0.368 | | 0.189 | |

CI, confidence interval; OR, odds ratio.

[a] Model 1 was adjusted for gender and age (month; continuous).

[b] Model 2 was adjusted for gender, age (month; continuous), health literacy score (tertile categories: ≤18 points, 19–20 points, ≥21 points, missing), time affluence (more affluent, neither, less affluent, non-affluent, missing).
